# A New Era for *Depression and Anxiety*: Global Perspective and Meaningful Impact

**DOI:** 10.1155/da/9860293

**Published:** 2025-09-04

*Letter from the Editor in Chief*

As *Depression and Anxiety* starts a new chapter, we are taking the time to reflect on where we have come from—and where we are heading. Under the previous leadership of Editor-in-Chief Murray Stein in collaboration with the Anxiety and Depression Association of America (ADAA) and Editor-in-Chief Peter P. Roy-Byrne the journal has been taking the toll of the challenges that mental health has faced in the last 10 years or so. We thank the Editors for the work they have done.

Under a new direction, the journal's mission will be to publishing work that truly resonates with and benefits our global audience. *Depression and Anxiety* will embrace a more international and interdisciplinary approach. We want to publish high-quality research and thoughtful reviews that speak to the real-world challenges of mental health. By fostering dialog and engagement across borders and disciplines, we are building a collaborative and diverse community that recognizes the multifaceted aspect of mental health. Social, political, and environmental forces all shape our well-being in complex ways. That is why we are prioritizing work that takes an inclusive, interdisciplinary approach to the understanding and treatment of depression and anxiety. Neurobiology, epidemiology, experimental psychopathology, pharmacology, and treatment innovations for mood and anxiety disorders will all fall within the scope of the journal. Some examples of topics our journal is interested in include: environmental degradation and its impact on mental health; political conflicts and mental health; new treatments for anxiety and depression new drugs development; mental health in adolescents and young adults, including how to preserve their well-being and treat any disorders; mental health in marginalized populations, including prisoners, ethnic minorities, LGBTQ+, people with disabilities, people who live with addictions, and people who live in extremely poor conditions; how our diet and nutrition is linked to some mental health disorders. We actively welcome contributions from clinicians, researchers, and individuals around the world who can offer a novel evidence-based approach. Studies that aim to replicate prior findings, while valuable, will be referred to our open-access partner journal *Brain and Behavior*, with a guarantee of peer review, to ensure fit and relevance.

We invite academics, clinicians, policymakers, and the public with lived experience to join us. Through shared insights, innovative research, and open exchange, we believe *Depression and Anxiety* can be more than a journal—it can be a catalyst for meaningful, global change in the field of mental health.

*Depression and Anxiety*

